# Homœopathic Therapeutics, as Applied to Obstetrics

**Published:** 1881-11

**Authors:** 


### BOOK NOTICES, REVIEWS, ETC.

HOMŒOPATHIC THERAPEUTICS, as applied to OBSTETRICS. By *Sheldon Leavitt, M. D.,* Prof. of Physiology and Clinical Midwifery in the Hahnemann Medical College and Hospital, Chicago. pp. 121, price $1.00? Chicago; Duncan Brothers. 1881.

This new advocate for professional favor claims that, only "the most *characteristic* features of a limited number of remedies, and those only, are embraced" in its design. In pursuance of this object, there is given the condensed symtomatology of ninety odd remedies, with a repertory. The indications for the remedies are short but in the main good; the repertory will be found especially useful. This brochure will be of service to those who are in the habit of searching for the *simillimum;* to all such we recommend it.

